# Impact of Kidney Failure on the Severity of COVID-19

**DOI:** 10.3390/jcm10092042

**Published:** 2021-05-10

**Authors:** Dorota Zarębska-Michaluk, Jerzy Jaroszewicz, Magdalena Rogalska, Beata Lorenc, Marta Rorat, Anna Szymanek-Pasternak, Anna Piekarska, Aleksandra Berkan-Kawińska, Katarzyna Sikorska, Magdalena Tudrujek-Zdunek, Barbara Oczko-Grzesik, Beata Bolewska, Piotr Czupryna, Dorota Kozielewicz, Justyna Kowalska, Regina Podlasin, Krzysztof Kłos, Włodzimierz Mazur, Piotr Leszczyński, Bartosz Szetela, Katarzyna Reczko, Robert Flisiak

**Affiliations:** 1 Department of Infectious Diseases, Jan Kochanowski University, 25-317 Kielce, Poland; reczko.katarzyna@poczta.fm; 2 Department of Infectious Diseases and Hepatology, Medical University of Silesia, 40-055 Katowice, Poland; jjaroszewicz@sum.edu.pl (J.J.); bgrzesik@hoga.pl (B.O.-G.); 3 Department of Infectious Diseases and Hepatology, Medical University of Białystok, 15-089 Białystok, Poland; pmagdar@gmail.com (M.R.); robert.flisiak1@gmail.com (R.F.); 4 Pomeranian Center of Infectious Diseases, Department of Infectious Diseases, Medical University of Gdańsk, 80-210 Gdańsk, Poland; lormar@gumed.edu.pl; 5 Department of Infectious Diseases and Hepatology, Wrocław Medical University, 50-367 Wrocław, Poland; marta.rorat@gmail.com (M.R.); aszymanek7@gmail.com (A.S.-P.); 6 Department of Forensic Medicine, Wrocław Medical University, 50-367 Wrocław, Poland; 7 Department of Infectious Diseases and Hepatology, Medical University of Łódź, 90-549 Łódź, Poland; annapiekar@gmail.com (A.P.); aleksandra.berkan@gmail.com (A.B.-K.); 8 Department of Tropical and Parasitic Diseases, Medical University of Gdańsk, 80-210 Gdańsk, Poland; ksikorska@gumed.edu.pl; 9 Department of Infectious Diseases and Hepatology, Medical University of Lublin, 20-059 Lublin, Poland; magdalena.tudrujek@gmail.com; 10 Department of Infectious Diseases, University of Medical Sciences, 61-701 Poznań, Poland; bbolewska@ump.edu.pl; 11 Department of the Infectious Diseases and Neuroinfections, Medical University in Białystok, 15-089 Białystok, Poland; avalon-5@wp.pl; 12 Department of Infectious Diseases and Hepatology, Faculty of Medicine, Collegium Medicum in Bydgoszcz, Nicolaus Copernicus University, 87-100 Toruń, Poland; d.kozielewicz@wsoz.pl; 13 Department of Adults Infectious Diseases, Medical University of Warsaw, 02-091 Warsaw, Poland; jdkowalska@gmail.com; 14 Hospital of Infectious Diseases in Warsaw, 01-201 Warsaw, Poland; podlasin@zakazny.pl; 15 Department of Infectious Diseases and Allergology, Military Institute of Medicine, 04-141 Warsaw, Poland; kklos@wim.mil.pl; 16 Clinical Department of Infectious Diseases in Chorzów, Medical University of Silesia, 41-500 Katowice, Poland; wlodek.maz@gmail.com; 17 Department of Rheumatology and Osteoporosis, Jozef Strus Hospital in Poznań, 61-285 Poznań, Poland; piotr_leszczynski@wp.pl; 18 Department of Rheumatology, Rehabilitation and Internal Medicine, Poznan University of Medical Sciences, 61-701 Poznań, Poland; 19 Department of Infectious Diseases, Liver Diseases and Acquired Immune Deficiencies, Wroclaw Medical University, 50-367 Wrocław, Poland; bartoszetela@gmail.com

**Keywords:** kidney failure, SARS-CoV-2, COVID-19, mortality

## Abstract

Background: Patients with kidney failure are at an increased risk of progression to a severe form of coronavirus disease 2019 (COVID-19) with high mortality. The current analysis was aimed to assess the impact of renal failure on the severity of COVID-19 and identify the risk factors of the fatal outcome in this population. Methods: The analysis included patients from the SARSTer database, a national real-world study evaluating treatment for COVID-19 in 30 Polish centers. Data were completed retrospectively and submitted online. Results: A total of 2322 patients were included in the analysis. Kidney failure was diagnosed in 455 individuals (19.65%), of whom 373 presented moderate stage and 82 patients, including 14 dialysis individuals, presented severe renal failure. Patients with kidney failure were significantly older and demonstrated a more severe course of COVID-19. The age, baseline SpO_2_, the ordinal scale of 4 and 5, neutrophil and platelet count, estimated glomerular filtration rate, and C-reactive protein concentration as well as malignancy and arterial hypertension were the independent predictors of 28-day mortality in logistic regression analysis. Conclusions: Underlying kidney disease in patients with COVID-19 is among the leading factors associated with a higher risk of severe clinical presentation and increased mortality rate.

## 1. Introduction

Severe acute respiratory syndrome coronavirus 2 (SARS-CoV-2) has rapidly spread worldwide since it was first identified in December 2019 in Wuhan. Despite an unprecedented global public health effort, the outbreak became pandemic on 11 March 2020. After one year, more than 120 million affected people with nearly 3 million deaths globally due to coronavirus disease 2019 (COVID-19) were documented [1]. The clinical spectrum of SARS-CoV-2 infection ranges from asymptomatic through mild and moderate respiratory illness to critical life-threatening viral pneumonia with respiratory failure, septic shock, and multiple organ dysfunction. The higher risk of the severe clinical presentation of COVID-19 is associated with older age, immunosuppressive therapy, and underlying comorbidities including cardiovascular and chronic pulmonary illnesses, diabetes, cancers, and chronic kidney diseases (CKD) [2,3,4].

The progressive loss of renal function in CKD results in alterations of the innate and adaptive immune system, including decreased leukocyte phagocytic activity, dwindling dendritic cells responsible for presenting antigens, depletion and dysfunction of B lymphocytes, and impaired cell-mediated immunity through an accelerated T cell turnover and increased apoptosis of cluster of differentiation (CD) 4+ and CD8+ lymphocytes [5]. The impaired immune response is associated with higher incidence and more severe course of infections which appear to be responsible for a large part of the mortality, especially in patients with end-stage renal disease (ESRD). Alongside secondary immunodeficiency, the immune activation in patients with chronic kidney disease is observed [6]. The increased production and decreased clearance of pro-inflammatory cytokines lead to systemic inflammation and oxidative stress, which contribute to atherosclerotic cardiovascular disease and other conditions worsening the prognosis of patients with SARS-CoV-2 infection.

The current analysis was aimed to assess the impact of kidney failure on the severity of COVID-19 and to identify the risk factors of the fatal outcome of the disease in this population in the real-world setting.

## 2. Materials and Methods

The study population consisted of patients included in the national database SARSTer, which is an ongoing project supported by the Polish Association of Epidemiologists and Infectiologists and covers 2784 adult individuals treated for COVID-19 between 1 March and 31 December 2020 in 30 Polish centers. All the patients were diagnosed with COVID-19 based on positive results of the real-time reverse transcriptase-polymerase chain reaction (RT-PCR) from the nasopharyngeal swab specimen [7].

The therapeutic management decisions were taken at the discretion of the treating physician following the current medical knowledge and in line with the national recommendations [8,9,10]. The SARSTer study had the approval of the Ethical Committee of the Medical University of Białystok with a granted waiver of informed consent from study participants due to its retrospective design, and the local bioethics committees in case of the off-label use of medication in patients with COVID-19.

Patients’ data were retrieved retrospectively from hospital files and completed online by a platform operated by “Tiba” sp. z o.o. The parameters gathered on admission included age, gender, body mass index (BMI), comorbidities and concomitant medications, clinical symptoms of SARS-CoV-2 infection, lung computed tomography scan, and selected lab values. The baseline laboratory data consisted of complete blood count, inflammatory indicators (C-reactive protein (CRP), procalcitonin (PCT), ferritin, and interleukin 6 (IL-6) concentration if tocilizumab (TCZ) prescription was considered), coagulation parameters such as D-dimer, international normalized ratio (INR), and fibrinogen, the activity of liver enzymes (aspartate and alanine aminotransferases, gamma-glutamyl transpeptidase, lactate dehydrogenase), and renal function tests. Estimated glomerular filtration rate (eGFR) was calculated with the MDRD Study equation and, using this measure, CKD was defined as eGFR < 60 mL/min/1.73 m^2^ along with a history of kidney disease from medical records [11]. According to renal function on admission, patients were stratified into three groups: eGFR < 30 mL/min/1.73 m^2^, eGFR 30–60 mL/min/1.73 m^2^, and eGFR > 60 mL/min/1.73 m^2^.

The COVID-19 severity on hospital admission was determined based on blood oxygen saturation (SpO_2_) and clinical status was defined as symptomatic stable with SpO_2_ > 95%, symptomatic unstable with two levels of baseline saturation SpO_2_ 91–95% or SpO_2_ ≤ 90%, and critical with acute respiratory distress syndrome (ARDS). The information on the medications applied for the treatment of COVID-19, including remdesivir (RDV), tocilizumab (TCZ), dexamethasone, convalescent plasma, low weight molecular heparin, and antibiotics, as well as drug-related adverse events, were collected during the hospitalization.

The patients were scored at baseline and then every 7 days during the following 28 days after admission on an ordinal scale, which includes eight categories: 1. unhospitalized, no activity restrictions; 2. unhospitalized, no activity restrictions and/or requiring oxygen supplementation at home; 3. hospitalized, does not require oxygen supplementation and does not require medical care; 4. hospitalized, requiring no oxygen supplementation, but requiring medical care; 5. hospitalized, requiring normal oxygen supplementation, low-flow by mask or nasal prongs; 6. hospitalized, on non-invasive ventilation with high-flow oxygen equipment; 7. hospitalized, for invasive mechanical ventilation or ECMO; 8. death.

The study outcomes included death, need for mechanical ventilation, and clinical improvement defined as at least a 2-point decrease in an ordinal scale classification from baseline to 14, 21, and 28 days of hospitalization.

To evaluate the impact of chronic kidney disease on the outcome of COVID-19, the analysis was performed concerning the eGFR at baseline.

### Statistical Analysis

The results are expressed as mean ± standard deviation (SD) or *n* (%) and odds ratios with 95% confidence intervals. *p* values of <0.05 were considered to be statistically significant. The significance of difference was calculated by Fisher’s exact test for nominal variables and by Mann–Whitney U and Kruskal–Wallis ANOVA for continuous and ordinal variables. Due to the highly variable group size, the Fisher’s *p*-values were accompanied by OR as the sample size independent effect size measures. The association between variables was measured by Spearman’s rank correlation coefficient and its significance test *p*-values. Survival analyses between patients with different eGFR ranges (Kaplan–Meier curves) were performed by Log-rank (Mantel–Cox) Test. Forward stepwise logistic regression models with Bayesian Information Criterion (BIC) as a model selection criterion were performed with death within 28-days after the start of hospitalization as the dependent variable. Among independent variables tested for the best model were age, sex, BMI, arterial hypertension, diabetes, coronary artery disease, chronic obstructive pulmonary disease, malignancy, GFR range, baseline levels of SpO2, CRP, procalcitonin, WBC, lymphocyte and neutrophil counts, platelets, D-dimer, ALT as well as therapy with dexamethasone, remdesivir, tocilizumab, and heparins. Logistic regression models were calculated by use of Statistica 13.0 (TIBCO Software Inc., Palo Alto, CA, USA).

## 3. Results

Among 2784 adult patients included in the SARSTer project, the data on kidney function were provided for 2322 individuals with a mean age of 60.4 ± 17.1 years and male predominance (53%). Among them, 455 individuals presented kidney impairment, a moderate stage of renal insufficiency was diagnosed in 373 patients with eGFR 30–60 mL/min/1.73 m^2^, of which six underwent kidney transplantation, and 82 patients with eGFR < 30 mL/min/1.73 m^2^ were diagnosed with severe renal failure (68 patients with non-dialysis dependent CKD) and ESRD (14 dialysis patients). Among patients with renal failure, 328 with moderate and 74 with severe stage had the diagnosis of chronic kidney disease based on the medical file, whereas in the remaining 53 individuals we were not able to confirm CKD due to incomplete records or disturbed communication with patients. Despite the lack of a previous diagnosis of CKD and no follow-up during three months after discharge from the hospital, we included these patients based on the depth-analysis of the available data concerning comorbidities and taking into account the age of patients as a risk factor of CKD and no improvement in the renal function after hydration. The detailed baseline characteristics of the patients according to kidney function on admission to the hospital are summarized in Table 1.

Patients with renal failure were significantly older and demonstrated a more severe course of COVID-19 on admission, defined by the higher rate of patients with an oxygen saturation ≤ 90% and a greater percentage of the more advanced categories on the ordinal scale. Patients with CKD more frequently suffered from diabetes and cardiovascular diseases including arterial hypertension, coronary artery disease, heart failure, and atrial fibrillation, and were more likely to be treated with insulin, oral antidiabetics, and antihypertensive drugs, compared to non-CKD individuals. Among medications reducing blood pressure, beta-blockers and diuretics were used predominantly in patients with renal failure (Supplementary Table S1).

Significantly higher values of inflammatory parameters including the concentration of CRP, PCT, and IL-6, as well as white blood cell and neutrophil counts, and lower platelet counts were documented in patients with CKD on admission (Table 2).

Patients with CKD were more often treated for COVID-19 with IL-6 inhibitor tocilizumab (TCZ), dexamethasone, and low molecular weight heparin compared with patients without kidney abnormalities. The application of remdesivir (RDV) was significantly lower in CKD patients, and five individuals with severe renal failure received off-label RDV, four of them were concurrently treated with TCZ, and two with dexamethasone—all were scored on admission in category 5 on the ordinal scale. Antibiotics were administered more frequently in those with severe renal failure (Table 3).

Continuous renal replacement therapy was continued only in 14 hemodialyzed patients, this therapy was not initiated by anyone else, and no patient was treated with hemoperfusion to remove cytokines.

As shown in Table 4, 28-day in-hospital mortality and the need for mechanical ventilation significantly increased in direct proportion to the degree of renal impairment. Moreover, clinical improvement was significantly slower in patients with advanced renal impairment.

The analysis of the outcome according to baseline kidney function that also takes into account the selected parameters at the admission is presented in the supplementary tables (Supplementary Tables S3–S5). 

The analysis performed depending on the survival revealed that patients who died were significantly older, with a higher proportion of males, a greater percentage of baseline oxygen saturation ≤ 90%, more severe clinical presentation on admission in terms of oxygen demand, more frequent comorbidities and treatment with concomitant medications, with significantly higher values of inflammatory parameters and D-dimer level, and lower platelet count. Those patients were more likely to be treated with TCZ, dexamethasone, convalescent plasma, low molecular weight heparin, and antibiotics. Furthermore, higher rates of individuals with moderate and severe kidney failure were reported in this group of patients (Table 5).

Among patients with eGFR < 30 mL/min/1.73 m^2^, no statistically significant differences were noticed between dialyzed and not dialyzed patients regarding baseline demographic and clinical measures as well as effectiveness outcomes (Supplementary Table S2). However, those who died were significantly older, with a higher proportion of baseline oxygen saturation ≤ 90%, were more likely to have coexisting conditions, with higher AST activity on admission, and were more frequently treated with antibiotics during hospitalizations (Table 6).

One of the five patients with eGFR < 30 mL/min/1.73 m^2^ who received RDV died due to sepsis. In the remaining four, no safety issues were observed and no deterioration in renal function was documented, and in two of them clinical improvement—in one after 21 and in another after 28 days of hospitalization—was reported.

**Figure 1:**
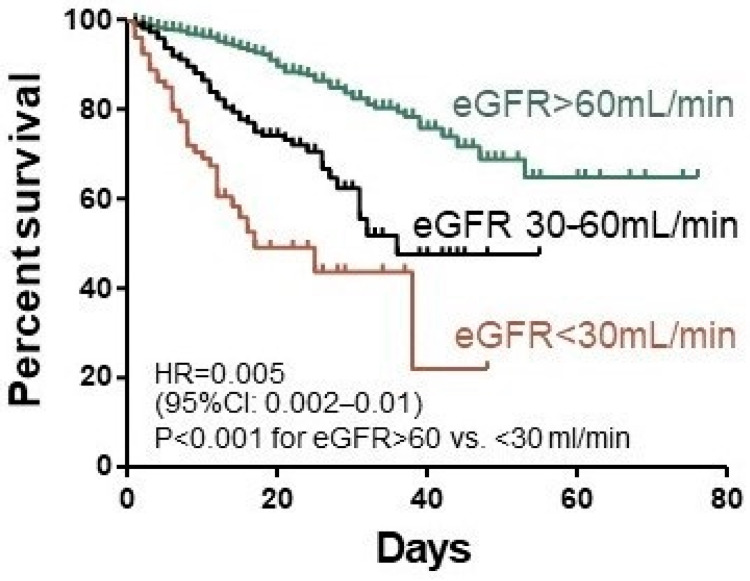
Kaplan–Meier survival curves of the CKD groups dependent on the eGFR.

The independent predictors of 28-day mortality in logistic regression analyses were age, baseline SpO2, the ordinal scale of 4 and 5, neutrophil and platelet count, eGFR, and CRP concentration (Figure 1, Table 7).

Among comorbidities, most notably malignancy as well as arterial hypertension (HA) and ischemic heart diseases were associated with mortality, while diabetes mellitus (DM) and chronic obstructive pulmonary disease (COPD) were not. Interestingly, in this cohort of 2322 COVID-19 patients, we were not able to show an independent effect of therapies, BMI, and baseline D-dimers, ALT, or procalcitonin on overall 28-day mortality.

## 4. Discussion

Chronic kidney disease is an increasing public health issue affecting 8–16% of the population worldwide [12]. Patients with CKD are highly susceptible to COVID-19 and are at an increased risk of progression to a severe or critical form of the disease because of impaired immunity; additionally, they are at enhanced risk of SARS-CoV-2 infection due to frequent hospital attendance [2,13,14,15,16,17,18]. The prevalence of CKD in patients with COVID-19 has been reported in a wide range of approximately 1–47%; however, it is suggested that the lowest values result from underestimation [16,19,20,21]. Among patients with COVID-19 hospitalized in 30 Polish centers included in the current analysis, nearly 20% were diagnosed with CKD, of which 18% presented severe renal failure. In addition to the worse patient’s status regarding the severity of COVID-19 when admitted to the hospital, we found the pre-existing renal disease to be independently associated with higher in-hospital mortality, especially in those with severe kidney failure, and our findings are in line with the results of previous studies [13,14,22,23,24,25,26]. The Global Burden of Disease (GBD) collaboration recently estimated the risk factors for severe COVID-19 worldwide using results from international databases and large multimorbidity studies from different countries, and determined that CKD is a condition conveying the highest risk for the severe presentation of the disease and COVID-19-related death [27]. In the current study, no difference in mortality was demonstrated between dialysis and non-dialysis dependent patients with severe renal failure, but it must be emphasized that the group of dialysis patients in the analyzed cohort was relatively small—only 14 patients. However, our findings of the comparable death risk regardless of the dialysis are consistent with observations from the study conducted by Flythe et al.

The reported death rates of 50% in 143 dialysis and 521 non-dialysis dependent individuals with CKD and 35% in 3600 non-CKD patients are higher than those noted in our analysis, but it is noteworthy that the abovementioned study included only critically ill patients with COVID-19 treated in intensive care units (ICU) [23]. The investigation performed by Yang et al. in 836 patients revealed in-hospital mortality rates of 9%, 50%, and 66.7% in non-CKD, non-dialysis dependent CKD, and dialysis patients, respectively. Of note, the proportions of individuals with the moderate presentation of COVID-19 were similar among those without and with non-dialysis dependent CKD (73.7% and 75%, respectively), and much lower in dialysis patients (40%), who were more frequently scored as severe cases on admission, which has had an impact on the fatal outcome [25].

The negative impact on the outcome was demonstrated for the baseline oxygen saturation corresponding to the severity of COVID-19 on admission—35.5% of patients with moderate and almost 56% with severe renal failure classified at baseline as SpO_2_ ≤ 90% died compared to a 16.5% mortality rate among non-CKD individuals. The category 5 in an ordinal scale on admission associated with the need for oxygen supplementation was an independent factor related to higher mortality, and our findings are consistent with the results of the other studies [28,29].

We confirmed older age as an independent strong predictor for in-hospital mortality, which is in line with previous reports and calculations performed by GBD collaboration in patients with CKD [2,3,15,22,23,27,30]. On the contrary, Cai et al., in a meta-analysis of 12 studies including CKD patients, documented a higher mortality rate in those below 70 years compared to older patients, explaining this finding by the more frequent rate of the other comorbidities with stronger than CKD association with increased risk of death among the elderly [14].

We did not demonstrate gender as a factor influencing the clinical status on admission and in-hospital mortality in CKD patients, and among those with severe renal failure, the death rate was nearly equal between females and males. Thus, the results of our study differ from other reports documenting a higher risk of severe course and COVID-19-related death rate in males [2,4,22,27,30,31].

In the current analysis, patients with CKD presented significantly increased baseline leukocyte and neutrophil counts, a higher level of inflammatory markers, including CRP, PCT, and IL-6 concentration, as well as D-dimer level compared to patients without CKD. Those parameters were increased in direct proportion to the degree of renal impairment and probably corresponded to a proinflammatory state. The high CRP concentration, the increased neutrophils, and the decreased platelet counts on admission were independently associated with a significantly higher in-hospital death rate in patients with CKD, which supported results from other studies [22,23,25,30,32,33]. Among comorbidities, arterial hypertension, ischemic heart disease, and malignancy were found to be independent negative predictors of 28-day survival and these findings, whereas diabetes and COPD were not. The impact of coexisting diseases was investigated in many studies and results are divergent depending on the analyzed population, sample size, and the nature of the study. Meta-analysis performed among Iranian patients by Merjalili et al. revealed that diabetes was associated with higher mortality, while arterial hypertension was not [26]. Park et al. analyzed the Korean population and found both diabetes and hypertension to be risk factors for death in the course of COVID-19 [34]. The same results were achieved by Gupta et al. among Indian patients [35]. Chen et al. demonstrated only ischemic heart disease and cerebrovascular disease to be independently associated with high mortality in Chinese patients [36]. According to the results of the meta-analysis conducted by Chaoqun et al., the presence of cerebrovascular disease, DM, COPD, malignancy, and hypertension was related to higher mortality in the course of COVID-19 [15]. Factors associated with an increased risk of death documented by Wiliamson et al. included ischemic heart disease, DM, and malignancy, especially hematological [2]. It should be pointed out that the percentage of diabetes among patients with severe renal failure was significantly higher compared to those with moderate kidney impairment and non-CKD individuals, which allows us to suppose that diabetic nephropathy is responsible for some cases of ESKD.

Of COVID-19-related medications, dexamethasone and tocilizumab were used more frequently in patients with CKD, probably as a result of more severe clinical presentation of the disease, so we were not able to show an independent effect of those therapies. According to the summary of product characteristics, the application of RDV was significantly lower in CKD patients; however, five individuals with severe renal failure scored at baseline in category 5 on an ordinal scale and received off-label RDV, and one of them died; however, it should be pointed out that in the remaining four patients no deterioration in renal function was documented [37]. Similar observations in a small series of 20 patients with ESKD treated with RDV were published by Pettit et al., where therapy appeared to be relatively safe and the potential benefit outweighed the theoretical risk of renal toxicity [38].

We are aware of several limitations of our study associated with its retrospective design—the analyses were based on the clinically available electronic captured data with possible entry errors, a lack of information about the causes of CKD, and in some cases a lack of confirmation of CKD due to incomplete records, and also missing laboratory tests in some patients, which did not allow us to assess the impact on the mortality of the selected parameters. As treatment with RDV and TCZ is not indicated in patients with eGFR <30 mL/min/1.73 m^2^ and, in turn, the absence of such treatment may influence the investigated outcomes, we may overestimate the causal effect of CKD on study endpoints. Lastly, due to the lack of control laboratory tests during hospitalization in some patients, we performed analysis taking into consideration the baseline renal status of patients with CKD, not looking at the development of acute kidney injury (AKI) in the course of COVID-19 in those individuals, although the experience from previous reports showed that AKI is associated with higher mortality [24,39,40,41].

However, the strengths of our study include a large number of patients from the heterogeneous population, covering different parts of the country, which increases the generalizability of the findings—all of them had a laboratory-confirmed SARS-CoV-2 infection and the patients were followed up for 28 days.

## Figures and Tables

**Table 1:** Baseline characteristics of patients according to kidney function.

Characteristic	eGFR > 60 mL/min *n* = 1867	eGFR 30–60 mL/min *n* = 373	eGFR < 30 mL/min *n* = 82	*p*
Age				
Mean (SD)	57.1 (16.5)	73.4 (12.5)	76.5 (12.9)	<0.001
>70 years (%)	397 (21.3)	240 (64.3)	57 (69.5)	<0.001
Gender				
Female, *n* (%)	869 (46.5)	177 (47.5)	44 (53.7)	0.44
Male, *n* (%)	998 (53.5)	196 (52.5)	38 (46.3)	
Body mass index, mean (SD)	27.8 (5.1)	28.5 (5.3)	29.2 (6.9)	0.03
Disease severity at the baseline, *n* (%)				
Oxygen saturation 91–95%	596 (31.9)	129 (34.6)	24 (29.3)	0.51
Oxygen saturation ≤ 90%	526 (28.2)	169 (45.3)	43 (52.4)	<0.001
Score on ordinal scale, *n* (%)				
3. Hospitalized, does not require oxygen supplementation and does not require medical care	131 (7%)	3 (1.9%)	1 (1.2%)	<0.001
4. Hospitalized, requiring no oxygen supplementation, but requiring medical care	833 (44.6)	108 (29)	21 (25.6)	<0.001
5. Hospitalized, requiring normal oxygen supplementation	835 (44.7)	244 (65.4)	54 (65.9)	<0.001
6. Hospitalized, on non-invasive ventilation with high-flow oxygen equipment	61 (3.3)	14 (3.7)	3 (3.7)	0.88
7. Hospitalized, for invasive mechanical ventilation or ECMO	6 (0.3)	0	3 (3.7)	-
Concomitant medications, *n* (%)	1071 (57.4)	331 (88.7)	69 (84.1)	<0.001
Coexisting conditions, *n* (%)	1285 (68.9)	354 (94.9)	77 (93.9)	<0.001
Arterial hypertension	719 (38.5)	268 (71.8)	53 (64.6)	<0.001
Coronary artery disease	155 (8.3)	92 (24.7)	27 (32.9)	<0.001
Heart failure	58 (3.1)	51 (13.7)	20 (24.4)	<0.001
Atrial fibrillation	88 (4.7)	59 (15.8)	11 (13.4)	<0.001
Diabetes	268 (14.4)	53 (14.2)	30 (36.6)	<0.001
Cerebrovascular disease	48 (2.6)	23 (6.2)	4 (4.9)	0.001
Malignancy	99 (5.3)	42 (11.3)	9 (11)	<0.001
Chronic obstructive pulmonary disease	46 (2.5)	29 (7.8)	2 (2.4)	<0.001
Bronchial asthma	91 (4.9)	20 (5.4)	6 (7.3)	0.58
Chronic liver disease	49 (2.6)	7 (1.9)	1 (1.2)	0.53
Dementia	47 (2.5)	21 (5.6)	6 (7.3)	0.001
Hypothyroidism	136 (7.3)	28 (7.5)	1 (1.2)	0.10

eGRF, estimated glomerular filtration rate; SD, standard deviation; ECM, extracorporeal membrane oxygenation.

**Table 2:** Baseline laboratory indicators according to the baseline kidney function.

Characteristic	eGFR > 60 mL/min *n* = 1867	eGFR 30–60 mL/min *n* = 373	eGFR < 30 mL/min *n* = 82	*p*
CRP mg/L, mean (SD)	65.5 (73.8)	91.7 (85.2)	107 (85.2)	<0.001
Procalcitonin ng/mL, mean (SD)	0.28 (1.82)	1.30 (6.8)	2.83 (6.6)	<0.001
Leukocytes 1/μL, mean (SD)	6405 (3079)	8962 (15028)	8700 (4563)	<0.001
Lymphocytes 1/μL, mean (SD)	1311 (909)	1532 (4064)	1026 (630)	<0.001
Neutrocytes 1/μL, mean (SD)	4446 (2767)	5717 (4575)	7050 (4136)	<0.001
Platelets 1000/μL, mean (SD)	221 (90.5)	202 (96)	208.5 (125.1)	<0.001
IL-6 pg/mL, mean (SD)	47.0 (94.2)	108.7 (209.1)	211.2 (600.3)	<0.001
D-dimers ng/mL, mean (SD)	1638 (5448)	2127 (3628)	5113 (11612)	<0.001
ALT IU/L, mean (SD)	41 (39)	36 (29)	52 (223)	0.001

CRP, C-reactive protein; ALT, alanine aminotransferase.

**Table 3:** In-hospital treatment for COVID-19 according to the baseline kidney function.

Medications	eGFR > 60 mL/min *n* = 1867	eGFR 30–60 mL/min *n* = 373	eGFR < 30 mL/min *n* = 82	*p*
Related to COVID-19, *n* (%)				
Remdesivir	454 (24.3)	81 (21.7)	5 (6.1)	<0.001
Tocilizumab	186 (9.9)	79 (21.1)	14 (17.1)	<0.001
Dexamethason	492 (26.3)	137 (36.7)	35 (42.7)	<0.001
Convalescent plasma	216 (11.6)	44 (11.8)	16 (19.5)	0.09
Low molecular weight heparin	1306 (70) *	299 (80.2) **	69 (84.1) ***	<0.001

* 1208 patients received prophylactic dose and 98 therapeutic dose. ** 251 patients were on prophylactic dose only, 17 received prophylactic dose on admission and then therapeutic dose during hospitalization, and 31 patients were on therapeutic dose from admission. *** 55 patients were on prophylactic dose, 14 patients received prophylactic then therapeutic and remaining 8 were on therapeutic dose.

**Table 4:** Outcome according to baseline kidney function.

	A eGFR > 60 mL/min	B eGFR 30–60 mL/min	C eGFR < 30 mL/min	Odds Ratio A vs. B	Odds Ratio B vs. C	Odds Ratio A vs. C
***n***	*n* = 1867	*n* = 373	*n* = 82			
Death, *n* (%)	132 (7.1)	82 (22)	35 (42.7)	0.27 (0.20–0.36) *p* < 0.001	0.38 (0.23–0.62) *p* < 0.001	0.10 (0.06–0.17) *p* < 0.001
Death time, mean (SD), days	14.4 (10.8)	10.8 (8.2)	8 (6.6)	<0.001	*p* = 0.01	*p* = 0.48
Mechanical ventilation, *n* (%)	86 (4.6)	35 (9.4)	10 (12.2)	0.47 (0.31–0.70) *p* < 0.001	0.74 (0.35–1.57) *p* = 0.42	0.35 (0.17–0.70) *p* = 0.006
Clinical improvement 14th day, *n* (%)	1068 (57.2)	158 (42.4)	21 (25.6)	1.81 (1.45–2.28) *p* < 0.001	2.13 (1.25–3.65) *p* = 0.006	3.89 (2.34–6.43) *p* < 0.001
Clinical improvement 21st day, *n* (%)	1467 (78.6)	222 (59.5)	34 (41.5)	2.45 (1.97–3.15) *p* < 0.001	2.07 (1.28–3.37) *p* = 0.003	5.18 (3.29–8.14) *p* < 0.001
Clinical improvement 28th day, *n* (%)	1601 (85.8)	262 (70.2)	40 (48.8)	2.55 (1.97–3.30) *p* < 0.001	2.47 (1.52–4.03) <0.001	6.32 (4.02–9.93) <0.001

**Table 5:** Comparison of patients who died or survived regardless of kidney function.

Characteristic	Died *N* = 249	28-Day Survive *N* = 2073	*p*-Value
Age			
Mean (SD)	74.2 (11.9)	58.7 (16.9)	<0.001
>70 years (%)	158 (63.5)	536 (25.9)	<0.001
Gender			
Female, *n* (%)	95 (38.2)	995 (48)	0.04
Male, *n* (%)	154 (61.8)	1078 (52)	0.04
Body mass index, mean (SD)	27.9 (6.1)	28 (5.1)	0.47
Disease severity at the baseline, *n* (%)			
Oxygen saturation 91–95%	51 (20.5)	698 (33.7)	<0.001
Oxygen saturation ≤ 90%	169 (67.9)	569 (27.5)	<0.001
Score on ordinal scale, *n* (%)			
3. Hospitalized, does not require oxygen supplementation and does not require medical care	1 (0.4)	138 (6.7)	<0.001
4. Hospitalized, requiring no oxygen supplementation, but requiring medical care	36 (14.5)	926 (44.7)	
5. Hospitalized, requiring normal oxygen supplementation	174 (69.9)	959 (46.3)	
6. Hospitalized, on non-invasive ventilation with high-flow oxygen equipment	30 (12)	48 (2.3)	
7. Hospitalized, for invasive mechanical ventilation or ECMO	8 (3.2)	1 (0.05)	
Concomitant medications, *n* (%)	205 (82.3)	1266 (61.1)	<0.001
Coexisting conditions, *n* (%)	233 (93.6)	1483 (71.5)	<0.001
Medication related to COVID-19, *n* (%)			
Remdesivir	61 (24.5)	479 (23.1)	0.68
Tocilizumab	55 (22.1)	224 (10.8)	<0.001
Dexamethason	135 (54.2)	529 (25.5)	<0.001
Convalescent plasma	50 (20.1)	226 (10.9)	<0.001
Low molecular weight heparin	203 (81.5)	1471 (71)	<0.001
Antibiotics	183 (73.5)	1045 (50.4)	<0.001
CRP mg/L, mean (SD)	128.5 (91.7)	64.2 (72.1)	<0.001
Procalcitonin ng/mL, mean (SD)	2.0 (6.2)	0.36 (2.98)	<0.001
Leukocytes 1/μL, mean (SD)	10,622 (16,729)	6450 (3963)	<0.001
Lymphocytes 1/μL, mean (SD)	1186 (2122)	1354 (1798)	<0.001
Neutrocytes 1/μL, mean (SD)	7354 (5150)	4441 (2796)	<0.001
Platelets 1000/μL, mean (SD)	210 (109)	219 (91)	0.008
IL-6 pg/mL, mean (SD)	192.4 (399.7)	50.2 (107.4)	<0.001
D-dimers ng/mL, mean (SD)	4654 (9820)	1507 (4722)	<0.001
ALT IU/L, mean (SD)	51 (133)	39 (37)	0.06
eGFR < 30 mL/min/1,73 m^2^, *n*(%)	35 (14.1)	47 (2.3)	<0.001
eGFR 30–60 mL/min/1,73 m^2^, *n*(%)	82 (32.9)	291 (13.7)	
eGFR > 60 mL/min/1,73 m^2^, *n*(%)	132 (53.0)	1735 (84.0)	

**Table 6:** Comparison of patients with eGFR < 30 mL/min who died or survived.

Characteristic	Died *N* = 35	28-Day Survive *N* = 47	*p*-Value
Age			
Mean (SD)	80.7 (9.4)	73.4 (14.2)	0.02
>70 years (%)	30 (85.7)	27 (57.4)	0.007
Gender			
Female, *n* (%)	19 (54.3)	25 (53.2)	1.00
Male, *n* (%)	16 (45.7)	22 (46.8)	1.00
Body mass index, mean (SD)	28.2 (7.7)	29.6 (6.5)	0.36
Disease severity at the baseline, *n* (%)			
Oxygen saturation 91–95%	7 (20)	17 (36.2)	0.14
Oxygen saturation ≤ 90%	24 (68.6)	19 (40.4)	0.01
Score on ordinal scale, *n* (%)			
3. Hospitalized, does not require oxygen supplementation and does not require medical care	0	1 (2.1)	1.00
4. Hospitalized, requiring no oxygen supplementation, but requiring medical care	7 (20)	14 (29.8)	0.44
5. Hospitalized, requiring normal oxygen supplementation	23 (65.7)	31 (66)	1.00
6. Hospitalized, on non-invasive ventilation with high-flow oxygen equipment	2 (5.7)	1 (2.1)	0.57
7. Hospitalized, for invasive mechanical ventilation or ECMO	3 (8.6)	0	0.07
Concomitant medications, *n* (%)	27 (77.1)	30 (63.8)	0.23
Coexisting conditions, *n* (%)	33 (94.3)	31 (66)	0.002
Medication related to COVID-19, *n* (%)			
Remdesivir	1 (2.9)	4 (8.5)	0.39
Tocilizumab	3 (8.6)	11 (23.4)	0.13
Dexamethason	15 (42.9)	20 (42.6)	1.00
Convalescent plasma	4 (11.4)	12 (25.6)	0.16
Low molecular weight heparin	28 (80)	41 (87.2)	0.54
Antibiotics	28 (65.1)	27 (57.5)	0.04
CRP mg/l, mean (SD)	120.1 (93)	97.3 (78.6)	0.28
Procalcitonin ng/mL, mean (SD)	4.75 (9.2)	1.45 (3.3)	0.07
Leukocytes 1/μL, mean (SD)	9351 (4540)	8214 (4568)	0.13
Lymphocytes 1/μL, mean (SD)	1042 (643)	1014 (628)	0.78
Neutrocytes 1/μL, mean (SD)	7803 (3871)	6527 (4274)	0.08
Platelets 1000/μL, mean (SD)	200 (103)	215 (141)	0.70
IL-6 pg/mL, mean (SD)	470.9 (1036.5)	95.8 (164.8)	0.24
D-dimers ng/mL, mean (SD)	4360 (4845)	5696 (14,940)	0.25
ALT IU/L, mean (SD)	86 (333)	26 (19)	0.43
AST IU/L, mean (SD)	72 (83)	38 (33)	0.03
GGTP IU/L, mean (SD)	33 (14)	69 (76)	0.60
LDH IU/L, mean (SD)	406 (205)	414 (192)	0.89
INR, mean (SD)	1.46 (0.83)	1.16 (0.14)	0.38
Fibrinogen mg/dL, mean (SD)	567 (150)	553.7 (216.1)	0.54
Ferritin mcg/L, mean (SD)	1828.2 (1507.3)	1244 (1533.7)	0.13

**Table 7:** Baseline factors independently associated with 28-days mortality based on forward stepwise logistic regression model.

	Estimate of β	SE	tStat	*p* Value
(Intercept)			854,282	<0.001
Age (per year)	0.139	0.023	5991	<0.001
SpO2 (%)	−0.213	0.025	−8578	<0.001
Neutrophils	0.153	0.022	6915	<0.001
Platelets	−0.073	0.020	−3655	<0.001
CRP (mg/dL)	0.048	0.022	2123	0.034
Ordinal scale (2)	−0.038	0.044	−0.857	0.391
Ordinal scale (3)	−0.055	0.042	−1302	0.193
Ordinal scale (4)	−0.160	0.081	−1987	0.047
Ordinal scale (5)	−0.195	0.080	−2429	0.015
Ordinal scale (6)	0.027	0.033	0.821	0.411
Arterial hypertension (no)	0.069	0.021	3260	0.001
Iscehmic heart disease (no)	−0.053	0.020	−2637	0.008
Malignancy (No)	−0.120	0.019	−6384	<0.001
eGFR < 30 mL/min	0.195	0.034	5649	<0.001
eGFR 30–60 mL/min	−0.090	0.034	−2592	<0.001

Chi^2 - statistic vs. constant model: 49.88, *p*-value < 0.001, BIC = 1119.

## Data Availability

Data supporting reported results can be provided upon request from the corresponding author.

## Supplementary Materials

The following are available online at https://www.mdpi.com/article/10.3390/jcm10092042/s1, Table S1: Medications for the treatment of comorbidities, Table S2: Patients with eGFR < 30 mL/min according to dialysis, Table S3: Patients with baseline CRP ≥ 100 mg/L—outcome according to kidney function, Table S4: Patients with baseline SpO2 ≤ 90%—outcome according to kidney function, Table S5: Patients with baseline D-dimers ≥ 1000 ng/mL—outcome according to kidney function.
