# Supplementary material for: Impact of Kidney Failure on the Severity of COVID-19
Source: J Clin Med. 2021 May 10;10(9):2042. doi: 10.3390/jcm10092042 (PMC8126240; doi:10.3390/jcm10092042)
Supplement: Supplementary file 1 [file jcm-10-02042-s001.zip › jcm-1182915-supplementary.pdf]

Impact of Kidney Failure on the Severity of COVID-19

Supplementary Table S1. Medications for the treatment of comorbidities.

Concomitant medications	eGFR > 60 mL/min *n* = 1867	eGFR 30-60 mL/min *n* = 373	eGFR < 30 mL/min *n* = 82
ACE blockers	264 (14.1)	86 (23.1)	13 (15.9)
Sartans	145 (7.8)	48 (12.9)	12 (14.6)
Beta blockers	341 (18.3)	123 (33.0)	35 (42.7)
Calcium channel blockers	174 (9.3)	60 (17.1)	18 (30)
Diuretics	160 (8.6)	91 (24.4)	28 (34.1)
Alpha blockers	27 (1.4)	12 (3.2)	2 (2.4)
Allopurinol	16 (0.9)	14 (3.8)	2 (2.4)
Statins	107 (5.7)	39 (10.5)	9 (11)
Insulin	56 (3)	25 (6.7)	11 (13.4)
Metformin	165 (8.8)	51 (13.7)	7 (8.5)
Other oral antidiabetic drugs	25 (1.3)	23 (6.2)	6 (7.3)
Oral anticoagulants	92 (4.9)	61 (17.4)	13 (15.9)
Acetylosalicylic acid	74 (4)	30 (8.0)	6 (7.3)
Steroids- systemic	31 (1.7)	17 (4.6)	2 (2.4)
Immunosupressive drugs	19 (1)	8 (2.1)	1 (1.2)
Proton pump inhibitors	33 (1.8)	16 (4.3)	0
Antidepressants	49 (2.6)	5 (1.3)	0
Antiepileptic drugs	35 (1.9)	5 (1.3)	1 (1.2)
Sedatives	14 (0,7)	2 (0.5)	0
Antipsychotic drugs	67 (3.6)	16 (4.3)	5 (6.1)

Supplementary Table S2. Patients with eGFR < 30 mL/min according to dialysis.

Characteristic	Dialysis patients *n* = 14	Patients with non-dialysis dependent CKD, *n* = 68	*P*-value
Age			
Mean (SD)	71.8 (9.5)	77.5 (13.3)	0.03
>70 years (%)	7 (50)	50 (73.5)	0.11
Gender			
Female, *n* (%)	7 (50)	37 (54.4)	0.78
Male, *n* (%)	7 (50)	31 (45.6)	0.78
Body mass index, mean (SD)	28.8 (7.8)	29.2 (6.8)	0.82
Disease severity at the baseline, *n* (%)			
oxygen saturation 91-95%	4 (28.6)	20 (29.4)	1.00
oxygen saturation ≤90%	6 (42.9)	37 (54.4)	0.56
Score on ordinal scale, *n* (%)			
4. hospitalized, requiring no oxygen supplementation, but requiring medical care	3 (21.4)	17 (25)	1.00
5. hospitalized, requiring normal oxygen supplementation	10 (71.4)	44 (64.7)	0.76
6. hospitalized, on non-invasive ventilation with high-flow oxygen equipment	0	3 (4.4)	1.00
7. hospitalized, for invasive mechanical ventilation or ECMO	0	3 (4.4)	1.00

Concomitant medications, *n* (%)	12 (85.7)	57 (83.8)	1.00
Coexisting conditions (>10% prevalence was included), *n* (%)	13 (92.9%)	64 (94.1)	1.00
arterial hypertension	7 (50%)	46 (67.6)	0.23
coronary artery disease	6 (42.9)	21 (30.9)	0.53
heart failure	4 (28.6)	16 (23.5)	0.73
atrial fibrillation	4 (28.6)	7 (10.3)	0.09
diabetes	3 (21.4)	27 (39.7)	0.24
malignancy	2 (14.3)	7 (10.3)	0.65
Medication-related to COVID-19, *n* (%)			
Remdesivir	0	5 (7.4)	0.58
Tocilizumab	1 (7.1)	13 (19.1)	0.44
Dexamethason	6 (42.9)	29 (42.6)	1.00
Covalescent plasma	4 (28.6)	12 (17.6)	0.46
Low molecular weight heparin	9 (64.3)	60 (88.2)	0.04
Death, n(%)	6 (42.9)	29 (42.6)	1.00
Mechanical ventilation, *n*(%)	0	10 (14.7)	0.20
Clinical improvement 14th day, *n*(%)	4 (28.6%)	17 (25)	0.75
Clinical improvement 21st day, *n*(%)	6 (42.9%)	28 (41.2)	1.00
Clinical improvement 28th day, *n*(%)	7 (50%)	33 (48.5)	1.00

Supplementary Table S3. Patients with baseline CRP ≥ 100 mg/L - outcome according to kidney function.

	**A eGFR > 60 mL/min**	**B eGFR 30-60 mL/min**	**C eGFR < 30 mL/min**	**Odds Ratio A vs. B**	**Odds Ratio B vs. C**	**Odds Ratio A vs. C**
n	*n* = 460	*n* = 120	*n* = 38			
Death, *n*(%)	73 (15.9%)	45 (37.5%)	17 (44.7%)	0.31 (0.20–0.49) P < 0.001	0.74 (0.35–1.55) P = 0.45	0.23 (0.11–0.46) P < 0.001
Mechanical ventilation, *n*(%)	58 (12.6%)	19 (15.8%)	4 (10.5%)	0.77 (0.43–1.35) P = 0.37	1.60 (0.51–5.04) P = 0.60	1.22 (0.42–3.58) P = 1.00
Clinical improvement 14th day, *n*(%)	202 (43.9%)	33 (27.5%)	8 (21.1%)	2.06 (1.33–3.21) P = 0.001	1.42 (0.59–3.42) P = 0.52	2.94 (1.31–6.54) P = 0.006
Clinical improvement 21st day, *n*(%)	315 (58.5%)	45 (37.5%)	13 (34.2%)	3.62 (2.38–5.51) P < 0.001	1.15 (0.54–2.48) P = 0.84	4.18 (2.08–8.40) P < 0.001
Clinical improvement 28th day, *n*(%)	350 (76.1%)	67 (55.8%)	17 (44.7%)	2.51 (1.65–3.83) P < 0.001	1.56 (0.75–3.25) P = 0.26	3.93 (2.00–7.71) <0.001

Supplementary Table S4. Patients with baseline SpO2 ≤ 90% - outcome according to kidney function.

	**A eGFR > 60 mL/min**	**B eGFR 30-60 mL/min**	**C eGFR < 30 mL/min**	**Odds Ratio A vs. B**	**Odds Ratio B vs. C**	**Odds Ratio A vs. C**
n	*n* = 526	*n* = 169	*n* = 43			

Death, *n*(%)	86 (16.5%)	60 (35.5%)	24 (55.8%)	0.35 (0.24–0.52) <0.001	0.44 (0.22–0.86) P = 0.02	0.15 (0.08–0.29) P < 0.001
Mechanical ventilation, *n*(%)	71 (13.5%)	29 (17.2%)	8 (18.6%)	0.75 (0.47–1.21) P = 0.26	0.91 (0.38–2.15) P = 0.82	0.68 (0.30–1.53) P= 0.36
Clinical improvement 14th day, *n*(%)	228 (43.3%)	48 (28.4%)	9 (20.9%)	1.93 (1.32–2.81) P<0.001	1.50 (0.67–3.36) P=0.44	2.89 (1.36–6.15) P=0.004
Clinical improvement 21st day, *n*(%)	349 (66.3%)	75 (44.4%)	12 (28.4%)	2.47 (1.74–3.52) P <0.001	2.06 (1.00–4.23) P =0.06	5.09 (2.55–10.16) P < 0.001
Clinical improvement 28th day, *n*(%)	396 (75.3%)	95 (56.2%)	14 (32.6%)	2.37 (1.65 – 3.42) P <0.001	2.66 (1.31 – 5.39) P = 0.006	6.31 (3.23 – 12.31) P < 0.001

Supplementary Table S5. Patients with baseline D-dimers ≥ 1000 ng/mL - outcome according to kidney function.

	**A eGFR > 60 mL/min**	**B eGFR 30–60 mL/min**	**C eGFR < 30 mL/min**	**Odds Ratio A vs. B**	**Odds Ratio B vs. C**	**Odds Ratio A vs. C**
n	*n* = 530	*n* = 186	*n* = 50			
Death, *n*(%)	76 (14.3%)	56 (30.1%)	25 (50%)	0.39 (0.26–0.58) P < 0.001	0.43 (0.23–0.81) P = 0.01	0.17 (0.09–0.31) P < 0.001
Mechanical ventilation, *n*(%)	38 (7.2%)	24 (12.9%)	7 (14%)	0.52 (0.30–0.90) P = 0.02	0.91 (0.38–2.25) P = 0.81	0.47 (0.20–1.12) P = 0.09
Clinical improvement 14th day, *n*(%)	251 (47.4%)	69 (37.1%)	14 (28%)	1.52 (1.08–2.15) P = 0.02	1.51 (0.76–3.01) P = 0.25	2.31 (1.22–4.39) P = 0.01
Clinical improvement 21st day, *n*(%)	370 (69.8%)	96 (51.6%)	19 (38%)	2.17 (1.54–3.05) P < 0.001	1.74 (0.92–3.30) P = 0.11	3.77 (2.07–6.88) P < 0.001
Clinical improvement 28th day, *n*(%)	408 (77%)	117 (62.9%)	22 (44%)	1.97 (1.38–2.83) P < 0.001	2.16 (1.15–4.06) P= 0.02	4.25 (2.35–7.71) P < 0.001
